# Osteoblasts induce glucose-derived ATP perturbations in chondrocytes through noncontact communication: Osteoblasts induce glucose-derived ATP perturbations in chondrocytes

**DOI:** 10.3724/abbs.2022042

**Published:** 2022-05-05

**Authors:** Jieya Wei, Yueyi Yang, Daimo Guo, Siqun Xu, Hongcan Huang, Demao Zhang, Jing Xie, Xuedong Zhou

**Affiliations:** 1 State Key Laboratory of Oral Diseases West China Hospital of Stomatology Sichuan University Chengdu 610041 China; 2 Department of Cariology and Endodontics West China Hospital of Stomatology Sichuan University Chengdu 610041 China

**Keywords:** adenosine triphosphate, carbon metabolism, chondrocyte, co-culture, osteoblast

## Abstract

Cartilage and subchondral bone communicate with each other through material and signal exchanges. However, direct evidence provided by experimental studies on their interactions is insufficient. In the present study, we establish a noncontact co-culture model with a transwell chamber to explore the energetic perturbations in chondrocytes influenced by osteoblasts. Our results indicate that osteoblasts induce more ATP generation in chondrocytes through an energetic shift characterized by enhanced glycolysis and impaired mitochondrial tricarboxylic acid cycle. Enhanced glycolysis is shown by an increase of secreted lactate and the upregulation of glycolytic enzymes, including glucose-6-phosphate isomerase (Gpi), liver type ATP-dependent 6-phosphofructokinase (Pfkl), fructose-bisphosphate aldolase C (Aldoc), glyceraldehyde-3-phosphate dehydrogenase (Gapdh), triosephosphate isomerase (Tpi1), and phosphoglycerate kinase 1 (Pgk1). Impaired mitochondrial tricarboxylic acid cycle is characterized by the downregulation of cytoplasmic aspartate aminotransferase (Got1) and mitochondrial citrate synthase (Cs). Osteoblasts induce the activation of Akt and P38 signaling to mediate ATP perturbations in chondrocytes. This study may deepen our understanding of the maintenance of metabolic homeostasis in the bone-cartilage unit.

## Introduction

Osteoarthritis (OA), a frequently observed degenerative joint disease, is characterized by articular cartilage loss, subchondral sclerosis, osteophyte formation, synovial inflammation, etc. [1]. Many recent studies on OA have focused on bone-cartilage crosstalk through paracrine events [ 2, 3]. Within the bone-cartilage unit, noncontact communication between chondrocytes and osteoblasts is allowed by vessels, microcracks, fissures, and uncalcified cartilage patches [ 4– 6]. This communication is further enhanced in OA due to impaired osteochondral integrity, increased porosity, prominent vascularization, and aggravated intraosseous hypertension [6].

Articular cartilage provides chondrocytes with a microenvironment that is hypocellular, avascular, and alymphatic, in which limited nutrients obtained from synovial fluid and subchondral bones are used to maintain the delicate balance between anabolism and catabolism. Glucose is the main metabolic substrate for adenosine triphosphate (ATP) production in chondrocytes, and it is metabolized through carbon metabolism constituted by glycolysis, the tricarboxylic acid (TCA) cycle, and the pentose phosphate pathway (PPP). PPP is a carbon utilization pathway that produces intermediate metabolites shared with glycolysis without generating or consuming ATP [7]. Glycolysis and the TCA cycle are two pathways related to the flux of glucose-derived carbons for ATP production.

First, glucose is converted to pyruvate via a series of glycolytic enzymes in the cytoplasm, including glucose-6-phosphate isomerase (Gpi), liver type ATP-dependent 6-phosphofructokinase (Pfkl), fructose-bisphosphate aldolase C (Aldoc), glyceraldehyde-3-phosphate dehydrogenase (Gapdh), triosephosphate isomerase (Tpi1), and phosphoglycerate kinase 1 (Pgk1) [ 8, 9]. Then pyruvate is transformed into lactate to complete the glycolysis steps under anaerobic or anoxic conditions or enters mitochondria for the TCA cycle and oxidative phosphorylation when there is a sufficient supply of oxygen [7]. The TCA cycle occurs in the mitochondrial matrix, and mitochondrial citrate synthase (Cs) is the entry enzyme into the TCA cycle [10]. The end products of the TCA cycle include a certain amount of ATP, reduced nicotinamide adenine dinucleotide (NADH), and flavin adenine dinucleotide, reduced (FADH _2_). NADH and FADH _2_ then transfer electrons for oxidative phosphorylation in the inner mitochondrial membrane. The flux that occurs with the TCA cycle and oxidative phosphorylation generates approximately 36 ATP molecules per glucose molecule in total, while the flux that occurs with glycolysis produces only 2 ATP molecules per glucose molecule [11]. Unexpectedly, although glycolysis is considered to be an energy-inefficient process, chondrocytes rely primarily on glycolysis for ATP production, even under normoxic conditions. The TCA cycle and oxidative phosphorylation only produce up to one-fourth of the total ATP [12]. However, the role of metabolic flux through the TCA cycle and oxidative phosphorylation cannot be underestimated, since they contribute to the redox balance and the activation and stabilization of glycolytic enzymes [13].

An increasing number of studies have been carried out on the physiological or pathological metabolic interactions between chondrocytes and osteoblasts. The initial study indicated that conditioned medium from OA osteoblasts facilitated glycosaminoglycan release in normal cartilage [14]. Subsequent studies revealed that intercellular communication induced changes in gene expression which are associated with hypertrophic differentiation, matrix mineralization, vascular invasion, and cartilage degradation [ 15– 17]. Various soluble factors were recognized to regulate metabolic interactions between chondrocytes and osteoblasts through signaling pathways such as the TGF-β/Smad [18], MAPK [19], and Wnt pathways [ 20, 21].

However, whether the energy metabolism of chondrocytes is affected by osteoblasts remains largely unknown. In this study, chondrocytes and osteoblasts were separated with a transwell insert to establish a noncontact co-culture model. Cells, cell lysates, or culture medium in control and osteoblast-induced groups were harvested to detect changes in glucose-derived ATP production in chondrocytes.

## Materials and Methods

### Cell culture

Animal studies were carried out according to ethical principles and all protocols were approved by the Institutional Review Board at the West China Hospital of Stomatology (WCHSIRB-D-2017-029) (Chengdu, China). Primary articular chondrocytes and calvarial osteoblasts were isolated from C57BL/6J mice (1–3 days old) purchased from Beijing HFK Bioscience Co., Ltd (Beijing, China). Briefly, after the mice were sacrificed, the epidermis was stripped with ophthalmic scissors under sterile condition to expose the knee joint and skull surfaces. The knee joints and skulls were collected and shredded into small pieces, washed three times with phosphate-buffered saline (PBS), and trypsinized in 0.25% protease solutions at 37°C for 30 min. Then, the supernatant containing trypsin was substituted with 0.5% type II collagenase for chondrocytes for 3 h or 0.5% type I collagenase for osteoblasts, followed by incubation for 1 h. Next, culture medium containing 10% heat-inactivated fetal bovine serum (FBS; HyClone, Logan, USA) and 1% penicillin-streptomycin (HyClone) was added into the mixture at 1:1 (v/v). The suspensions were centrifuged at 157 *g* for 5 min, and primary cells were collected into T25 flasks and cultured in a humidified atmosphere of 5% CO _2_ at 37°C for subsequent experiments.

### Transwell co-culture system construction

The transwell co-culture system was established as previously described [17]. Briefly, chondrocytes were seeded in 6-well plates or 35-mm glass bottom dishes, while osteoblasts were plated in 6-well transwell chambers with pore size of 0.4 μm. After 12 h of equilibration with medium containing 10% FBS, the cells were starved with 2% FBS medium for another 12 h. Then the culture medium was replaced by medium containing 1% FBS, transwell inserts were transferred to corresponding wells for co-culture. Chondrocytes cultured in the absence of osteoblasts served as the control group. At the indicated time points, cells, cell lysates, and culture medium were harvested from the lower chambers.

### Measurement of intracellular ATP level

Intracellular ATP levels of chondrocytes were detected using the Enhanced ATP Assay kit (Beyotime Institute of Biotechnology, Shanghai, China) according to the manufacturer’s instructions. Briefly, after 3 days of co-culture, chondrocytes were washed three times with cold PBS and immediately lysed on ice. The cell lysates were centrifuged at 12,000 *g* and 4°C for 5 min. Then, 20 μL supernatant and 100 μL detection working solution were added, mixed gently, and incubated for 5 min in each well of a 96-well plate. Next, the luminescence levels were measured with a Synergy HTX Multi-Mode Microplate Reader (BioTek Instruments, Winooski, USA) within 10 min.

### Detection of lactate secretion

Lactate secretion levels were detected using a Lactate Detection kit (Jiancheng Biotechnology, Nanjing, China). Chondrocyte culture medium (100 μL) was collected at 24, 48, 72, and 96 h after co-culture, and added to each well of a 96-well plate. After 10 min of incubation with the working solution and chromogenic agent at 37°C, 100 μL stop solution was added into each well. The absorbance levels were detected at a wavelength of 530 nm with a Multiskan GO microplate spectrophotometer (Thermo Fisher Scientific, Waltham, USA).

### Supravital staining of mitochondria

After 3 days of co-culture, live chondrocytes were washed three times with warm PBS and stained with the Cell Navigator™ Mitochondrion Staining kit (Red Fluorescence; AAT Bioquest, Sunnyvale, USA) in a humidified incubator with 5% CO _2_ at 37°C for 2 h. Then, the Mitolite™ Red working solution was replaced by warm PBS mixed with growth medium at a 1:1 (v/v). A fluorescence microscope with a Texas Red ^®^ filter set (Ex/Em=585/610 nm) was used to examine the mitochondria in chondrocytes.

### RNA sequencing

After 3 days of co-culture, total RNAs were isolated from cells using Trizol (Thermo Fisher Scientific) and sent to Shanghai Lifegenes Biotechnology Co., Ltd (Shanghai, China) for transcriptome analysis. Briefly, RNA integrity was assessed and 1.5 μg RNA was used for each sample. The HiSeq 4000 PE Cluster kit (Illumina, San Diego, USA) was used for clustering. Clean data were acquired from raw data of fast q format through in-house Perl scripts. Then, these data were aligned to the reference genome with HISAT2 v2.1.0 and analyzed with HTSeq v0.6.1. For the differentially expressed genes, Gene Ontology enrichment analysis, Kyoto Encyclopaedia of Genes and Genomes enrichment analysis and differential expression analysis were performed. Significance was set at *P*<0.05 and |foldchange|≥1.5.

### Quantitative real-time polymerase chain reaction (qRT-PCR)

The mRNA expression levels of key enzymes in carbon metabolism were detected by qRT-PCR, including glucose-6-phosphate isomerase (Gpi), liver type ATP-dependent 6-phosphofructokinase (Pfkl), fructose-bisphosphate aldolase C (Aldoc), glyceraldehyde-3-phosphate dehydrogenase (Gapdh), triosephosphate isomerase (Tpi1), phosphoglycerate kinase 1 (Pgk1), and cytoplasmic aspartate aminotransferase (Got1). After 3 days of co-culture, chondrocytes were washed twice with ice-cold PBS. RNA was isolated using the RNeasy Plus Mini kit (Qiagen, Valencia, USA) with a genomic DNA eliminator according to the manufacturer’s recommendation. RNA samples were dissolved in RNase-free water and quantitated by measuring the absorbance at 260 nm with a NanoDrop ^®^ spectrophotometer (Nano Spectrophotometer 2000c; Thermo Fisher Scientific). After treatment with DNase I (Thermo Fisher Scientific), RNA samples were reverse-transcribed into complementary DNAs using a cDNA synthesis kit (Thermo Fisher Scientific, USA). qRT-PCR was performed in a 25-μL volume system containing 0.5 μM primer pairs and 1 μL template cDNA using the SYBR Premix ExTaqII PCR Kit (TAKARA, Shiga, Japan) and the iCycler (Bio-Rad, Hercules, USA). After pre-incubation at 95°C for 5 min to activate the polymerase, the reaction went through 45 cycles of amplification. Each circle was sequentially composed of denaturation at 94°C for 15 s, annealing at 64°C for 15 s, and elongation at 72°C for 15 s. The relative expressions of all genes were determined using the 2 ^−ΔΔCt^ method. The housekeeping gene hypoxanthine-guanine phosphoribosyltransferase ( *Hprt*) served as the internal control for data normalization. Sequences of primer pairs used in this study are listed in Table 1.

**Table:** **Table1** Sequence of primers for qPCR in this study

Gene	Forward primer sequence (5′→3′)	Reverse primer sequence (5′→3′)	Product size (bp)
*Hprt*	TTGGGCTTACCTCACTGCTT	GCAAAAAGCGGTCTGAGGAG	72
*Gpi*	TTGTCGCCCTGTCTACGAAC	AGCTGCTCGAAGTGGTCAAA	160
*Pfkl*	CGCTGCAATGGAGAGTTGTG	CCTCAAAGACGTAGGCAGCA	152
*Aldoc*	CACCTTCTCCTATGGGCGTG	GATGTAGAGGGACTGTG	184
*Gapdh*	TCAAGCTCATTTCCTGGTATGAC	GGGATAGGGCCTCTCTTGCT	141
*Tpi1*	ACTCATGGTTGGAGCACAGG	AAGCTAGAGCCAAGGCCATC	74
*Pgk1*	CCTTTCGACCTCACGGTGTT	AGGAACGTTGAAGTCCACCC	110
*Got1*	AACGACAACAGCCTCAACCA	AAAGACTGCACCCCTCCAAC	134

Hprt, hypoxanthine-guanine phosphoribosyltransferase; Gpi, glucose-6-phosphate isomerase; Pfkl, liver type ATP-dependent 6-phosphofructokinase; Aldoc, fructose-bisphosphate aldolase C; Gapdh, glyceraldehyde-3-phosphate dehydrogenase; Tpi1, triosephosphate isomerase; Pgk1, phosphoglycerate kinase 1; and Got1, cytoplasmic aspartate aminotransferase.

### Western blot analysis

Chondrocytes in the control and osteoblast-induced groups were washed three times with cold PBS. Cell lysates were obtained using lysis buffer supplemented with the protease inhibitor phenylmethanesulfonyl fluoride (Sigma-Aldrich, St Louis, USA). BCA Protein Assay kit (Beyotime Institute of Biotechnology) was used to determine the protein concentration. Protein samples were separated by 10% sodium dodecyl sulfate-polyacrylamide gel electrophoresis and transferred onto polyvinylidene difluoride membranes (Millipore, Billerica, USA). Membranes were blocked with Tris-buffered saline containing 0.05% Tween 20 and 5% fat-free dry milk for 1 h at room temperature, and then incubated with primary antibodies (1:1000) overnight at 4°C, followed by incubation with the corresponding anti-rabbit (mouse anti-rabbit IgG-HRP; 1:2000; Santa Cruz Biotechnology) or anti-mouse (m-IgGλBP-HRP; 1:4000; Santa Cruz Biotechnology) secondary antibodies for 2 h at room temperature. Finally, immunoreactive bands were visualized using the Immobilon Western Chemilum HRP substrate (Millipore) and analyzed by ImageJ software (NIH, Bethesda, USA). Primary antibodies used in this study are anti-β-actin antibody (sc-47778; Santa Cruz Biotechnology, Dallas, USA), anti-Gpi antibody (ab66340; Abcam, Cambridge, UK), anti-Pfkl antibody (ab181064; Abcam), anti-Aldoc antibody (ab87122; Abcam), anti-Pgk1 antibody (ab199438; Abcam), anti-mitochondrial citrate synthase (Cs) antibody (383932; ZEN-BIOSCIENCE, Chengdu, China), anti-Akt antibody (342529; ZEN-BIOSCIENCE), anti-pAkt antibody (381555; ZEN-BIOSCIENCE), anti-P38 antibody (340697; ZEN-BIOSCIENCE), and anti-pP38 antibody (310091; ZEN-BIOSCIENCE).

### Confocal laser-scanning microscopy (CLSM)

After being seeded into petri dishes specified for CLSM, chondrocytes were equilibrated for 12 h with medium containing 10% FBS and then starved with medium containing 2% FBS for another 12 h. The transwell co-culture system was established using medium supplemented with 1% FBS. At the indicated time points, chondrocytes were sequentially rinsed with warm PBS three times, fixed with cold 4% paraformaldehyde for 10 min, permeabilized in 0.25% Triton X-100 for 10 min, and blocked with 5% bovine serum albumin for 1 h. After incubation with primary antibodies (1:200) against proteins involved in carbon metabolism (including Gpi, Pfkl, Aldoc, Pgk1, and Cs) and several signaling pathways (including Akt, pAkt, P38, and pP38) overnight at 4°C, cells were incubated with Alexa Fluor 647-conjugated anti-rabbit IgG secondary antibody (1:200; Life Technology, Grand Island, USA) for 2 h at room temperature. Next, the cytoskeletons were stained with FITC-conjugated phalloidin (Invitrogen, Carlsbad, USA) overnight at 4°C, and the nuclei were stained with DAPI (Sigma-Aldrich) for 10 min at room temperature. Finally, images were captured with a confocal laser-scanning microscope (FV3000; Olympus, Tokyo, Japan) and further analyzed with Image-Pro Plus 6.0 software (Media Cybernetics, Bethesda, USA).

### Statistical analysis

At least three independent experiments were performed. The data were analyzed with GraphPad Prism 7.00 software (GraphPad Software Inc., San Diego, USA) using independent sample *t* test analysis or two-way analysis of variance to explore the statistical differences (set at *P*<0.05) between groups.

## Results

### Osteoblasts induce higher ATP levels, greater lactate secretion and fewer mitochondria in chondrocytes

**Figure 1:**
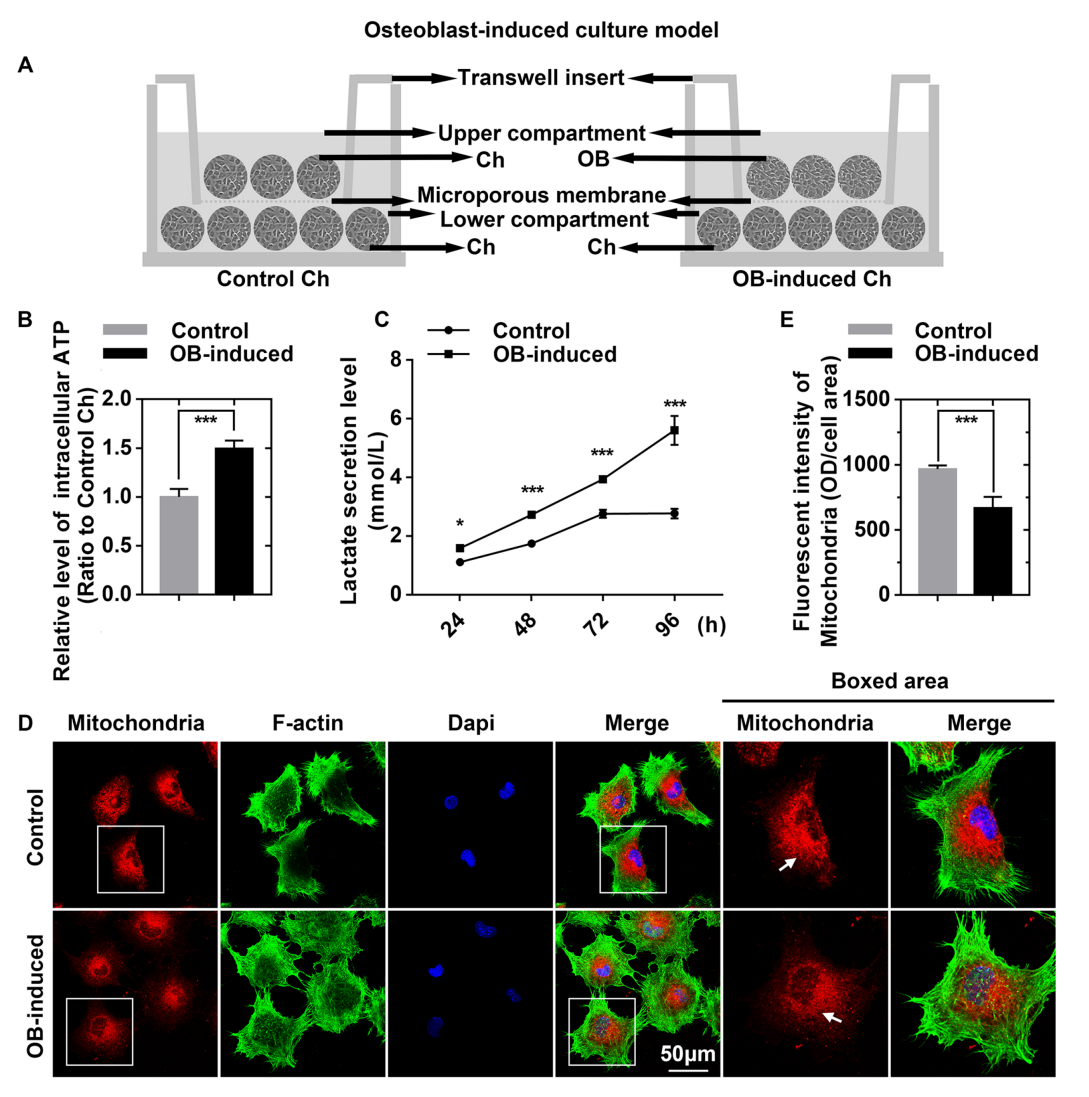
Figure1 Osteoblasts induce higher ATP levels, greater lactate secretion and fewer mitochondria in chondrocytes (A) Schematic diagram illustrating the co-culture model by which chondrocytes and osteoblasts communicate. (B) Intracellular ATP levels in control and osteoblast-induced chondrocytes measured using an enhanced ATP assay kit. Cell lysates were obtained after 3 days of co-culture. The luminescence levels were detected by a Synergy HTX Multi-Mode Microplate Reader in three independent experiments (n=3). (C) Lactate secretion levels were detected using the lactate detection kit. Chondrocyte culture medium was collected at 24, 48, 72, and 96 h after co-culture. The absorbance levels were detected with a Multiskan GO microplate spectrophotometer in three independent experiments (n=3). (D) Images of immunofluorescence staining showing the decrease in the number of mitochondria in chondrocytes. Mitochondria of live chondrocytes were stained with MitoLite™ Red (Red) using a Cell Navigator™ mitochondrion staining kit. After fixation with 4% PFA, the cytoskeletons were stained with FITC-phalloidin (green), and the nuclei were stained with DAPI (blue). CLSM images were obtained from three independent experiments (n=3). (E) Fluorescence quantification was performed to determine the average fluorescence intensity of mitochondria. The results were from three independent replicates (n=3). The data in B, C, and E are presented as the mean±SD. A significant difference was observed in the control chondrocytes. *P<0.05, **P<0.025, and ***P<0.01. Ch, chondrocytes; OB, osteoblasts.

Chondrocytes and osteoblasts were separated with a transwell insert to establish the noncontact co-culture model ( Figure 1A). The enhanced ATP assay kit, lactate detection kit and mitochondrial staining kit were applied to detect changes related to ATP production in chondrocytes. By using an enhanced ATP assay kit, we detected differences in luminescence levels and found that ATP production in chondrocytes induced by osteoblasts was higher (up to 1.496-fold) than that in control chondrocytes ( Figure 1B). By using a lactate detection kit, we found that there was more accumulation of secreted lactate in the osteoblast-induced chondrocyte group than in the control chondrocytes ( Figure 1C), which indicated that osteoblasts induced the enhancement of glycolysis in chondrocytes [ 22, 23]. Mitochondrial staining results showed decreased mitochondrial number in osteoblast-induced chondrocytes compared to that in the control chondrocytes ( Figure 1D), which indicated the impairment of oxidative phosphorylation of mitochondria [24]. Fluorescence quantification was further performed to determine the changes in mitochondrial number ( Figure 1E).

### Osteoblasts induce alterations in the mRNA expressions of genes related to glucose-derived ATP perturbations in chondrocytes

**Figure 2:**
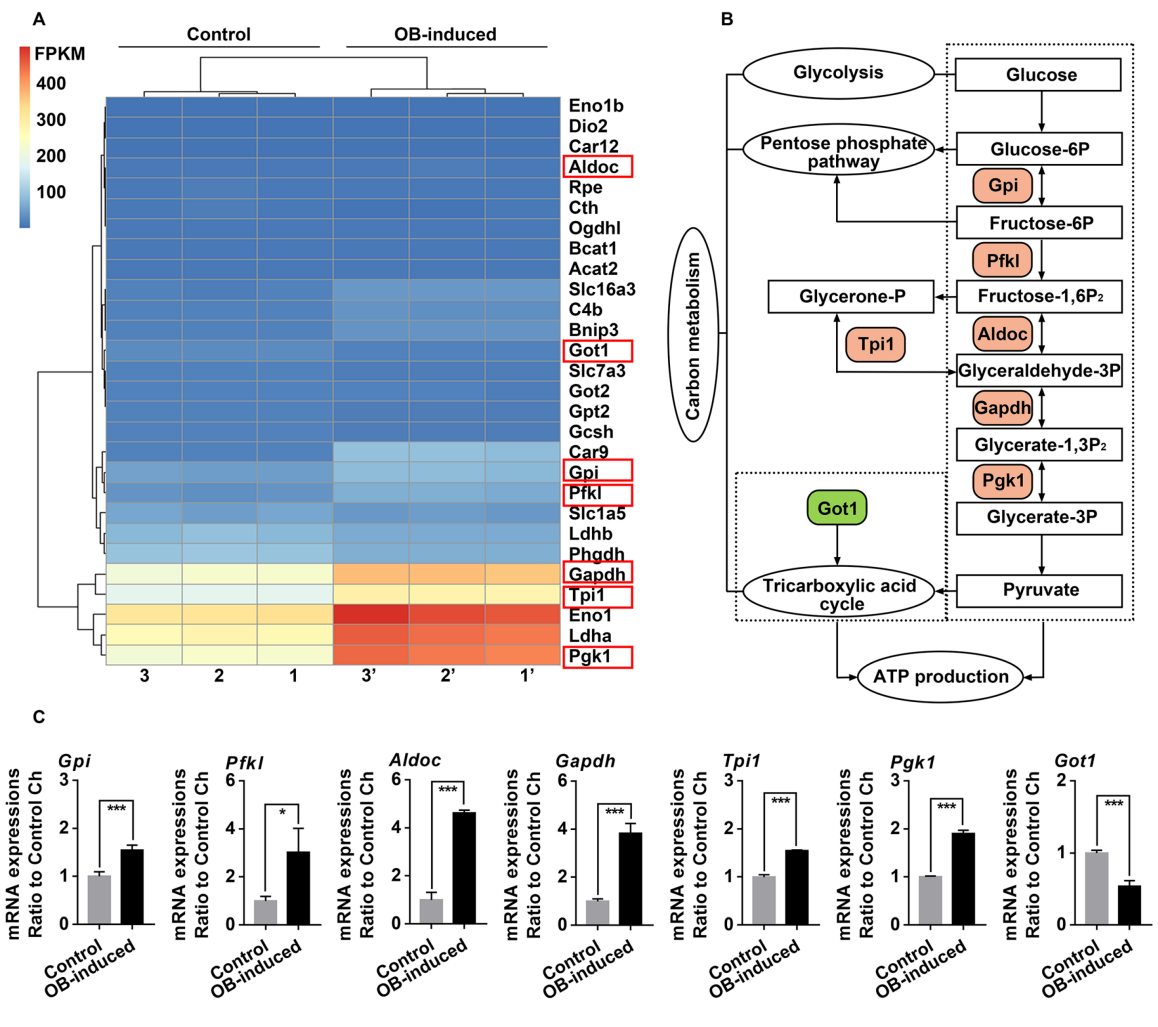
Figure2 Osteoblasts induce alterations in the mRNA expressions of genes related to glucose-derived ATP perturbations in chondrocytes (A) Heatmap from RNA sequencing showing the change of genes related to carbon metabolism pathway in chondrocytes after 3 days of co-culture with osteoblasts. Three pairs of samples, i.e., samples 1 and 1’, samples 2 and 2’, and samples 3 and 3’, were obtained from three independent mother cells (n=3). Genes in red boxes are significantly changed in terms of carbon metabolism. (B) Schematic diagram showing the crucial steps and key enzymes of the flux of glucose-derived carbons for ATP production in chondrocytes. Tree diagram was obtained from the online KEGG enrichment analysis. Color-labeled candidates are changed in osteoblast-induced chondrocytes in this study. (C) qRT-PCR confirmed the gene expression of key enzymes in the flux of glucose-derived carbons for ATP production in chondrocytes after 3 days of co-culture with osteoblasts. Six enzymes, including Gpi, Pfkl, Aldoc, Gapdh, Tpi1, and Pgk1, were upregulated, while Got1 was downregulated. Hprt was used as the internal control. mRNA expression is presented as the fold change ratio to the control chondrocytes. The data shown are representative of three independent experiments (n=3). The data in C are presented as the mean±SD. Significant differences were observed in the control chondrocytes. *P<0.05, **P<0.025, and ***P<0.01. Ch, chondrocytes; OB, osteoblasts.

To explore the changes of genes involved in the carbon metabolism pathway, RNA sequencing was performed in chondrocytes co-cultured with osteoblasts for 3 days, and Figure 2A showed all the changed gene candidates related to carbon metabolism. Compared with the carbon metabolism pathway provided by online KEGG enrichment analysis, gene candidates in the crucial steps of the flux of glucose-derived carbons for ATP production were identified ( Figure 2B). To further confirm these gene changes, we designed primers for these seven key enzymes, including glucose-6-phosphate isomerase (Gpi), liver type ATP-dependent 6-phosphofructokinase (Pfkl), fructose-bisphosphate aldolase C (Aldoc), glyceraldehyde-3-phosphate dehydrogenase (Gapdh), triosephosphate isomerase (Tpi1), phosphoglycerate kinase 1 (Pgk1), and cytoplasmic aspartate aminotransferase (Got1), and performed qRT-PCR ( Figure 2C). The results indicated that Gpi, Pfkl, Aldoc, Gapdh, Tpi1, and Pgk1 were upregulated (more than 1.550-fold in all members), while Got1 was downregulated (0.537-fold) in the osteoblast-induced group. The upregulation of Gpi, Pfkl, Aldoc, Gapdh, Tpi1, and Pgk1 and the downregulation of Got1 indicated the enhancement of glycolysis and impairment of oxidative phosphorylation of mitochondria, which might lead to ATP perturbations in chondrocytes at the gene level [ 8, 9, 25].

### Osteoblasts induce alterations in the protein expressions of key enzymes involved in glucose-derived ATP perturbations in chondrocytes

**Figure 3:**
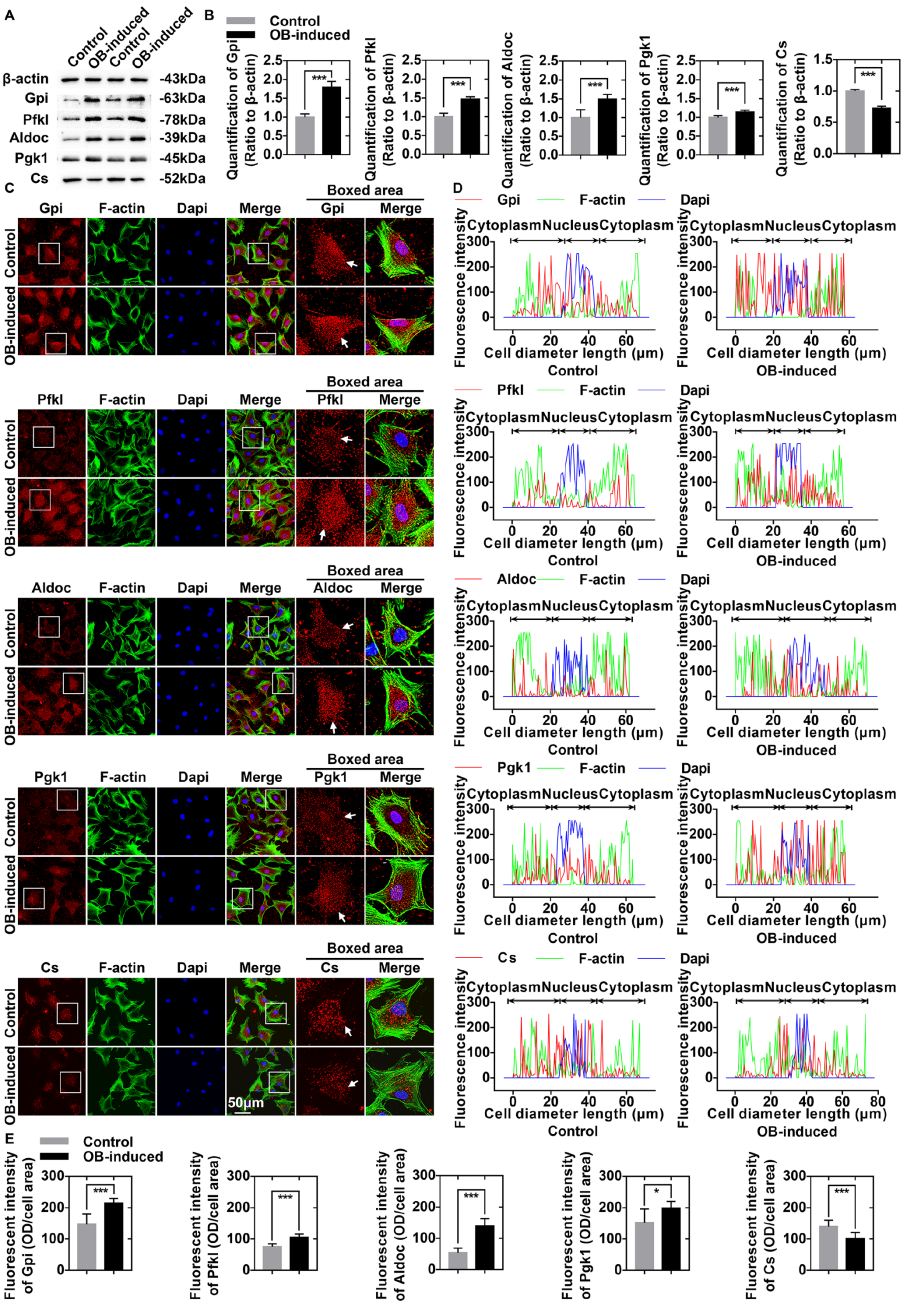
Figure3 Osteoblasts induce alterations in the protein expressions of key enzymes involved in glucose-derived ATP perturbations in chondrocytes (A) Western blots showing the protein expressions of Gpi, Pfkl, Aldoc, Pgk1, and Cs in osteoblast-induced chondrocytes. Cell lysates were harvested after 3 days of co-culture with osteoblasts. Three replicates were used, and the representative gels were further analysed (n=3). (B) Relative changes in Gpi, Pfkl, Aldoc, Pgk1, and Cs in (A) were confirmed by OD quantification. (C) Immunofluorescence staining of Gpi, Pfkl, Aldoc, Pgk1, and Cs (red) in chondrocytes after 3 days of co-culture with osteoblasts. Pfkl, Aldoc, and Cs were present mainly in the cytoplasm; Gpi and Pgk1 were present in the cytoplasm and nuclei. The expressions of Gpi, Pfkl, Aldoc, and Pgk1 in chondrocytes were increased after 3 days of co-culture with osteoblasts. However, the expression of Cs was decreased in osteoblast-induced chondrocytes (boxed area). The cytoskeletons were stained with FITC-phalloidin (green), and the nuclei were stained with DAPI (blue). The images captured by CLSM were obtained from three independent experiments (n=3). (D) Image-Pro Plus 6.0 was used to determine the linear fluorescence intensity and explore the distributions of Gpi, Pfkl, Aldoc, Pgk1, and Cs in (C). Data analysis was performed on at least 10 cells per group. (E) Fluorescence quantification was performed to determine the changes in Gpi, Pfkl, Aldoc, Pgk1, and Cs in (C). The results were from three independent replicates (n=3). The data in B and E are presented as the mean±SD. A significant difference was observed for control chondrocytes. *P<0.05, **P<0.025, and ***P<0.01. OB, osteoblasts.

We further performed western blot analysis to confirm the changes of proteins involved in the flux of glucose-derived carbons for ATP production ( Figure 3A). The results showed that the protein levels of Gpi (1.796-fold), Pfkl (1.474-fold), Aldoc (1.496-fold), and Pgk1 (1.14-fold) were upregulated, while the protein level of mitochondrial citrate synthase (Cs) (0.723-fold) was downregulated in chondrocytes after 3 days of co-culture with osteoblasts ( Figure 3B).

Then immunofluorescence staining was performed to explore the subcellular distributions of these key enzymes. The results indicated that the expressions of Pfkl and Aldoc were increased in the cytoplasm of osteoblast-induced chondrocytes. Gpi and Pgk1 were present both in the cytoplasm and in the nuclei, and their expressions were also increased in chondrocytes after 3 days of co-culture with osteoblasts. Cs was present mainly in the cytoplasm; however, we observed a decreased expression of Cs in the osteoblast-induced group ( Figure 3C). Fluorescence quantification confirmed the changes in Gpi, Pfkl, Aldoc, Pgk1, and Cs ( Figure 3D,E). The increased protein expressions of Gpi, Pfkl, Aldoc, and Pgk1 and decreased protein expression of Cs indicated the enhancement of glycolysis and impairment of oxidative phosphorylation of mitochondria, respectively, which might lead to ATP perturbations in chondrocytes at the protein level [ 8– 10].

### Osteoblasts regulate glucose-derived ATP production through the Akt and P38 signaling pathways in chondrocytes

**Figure 4:**
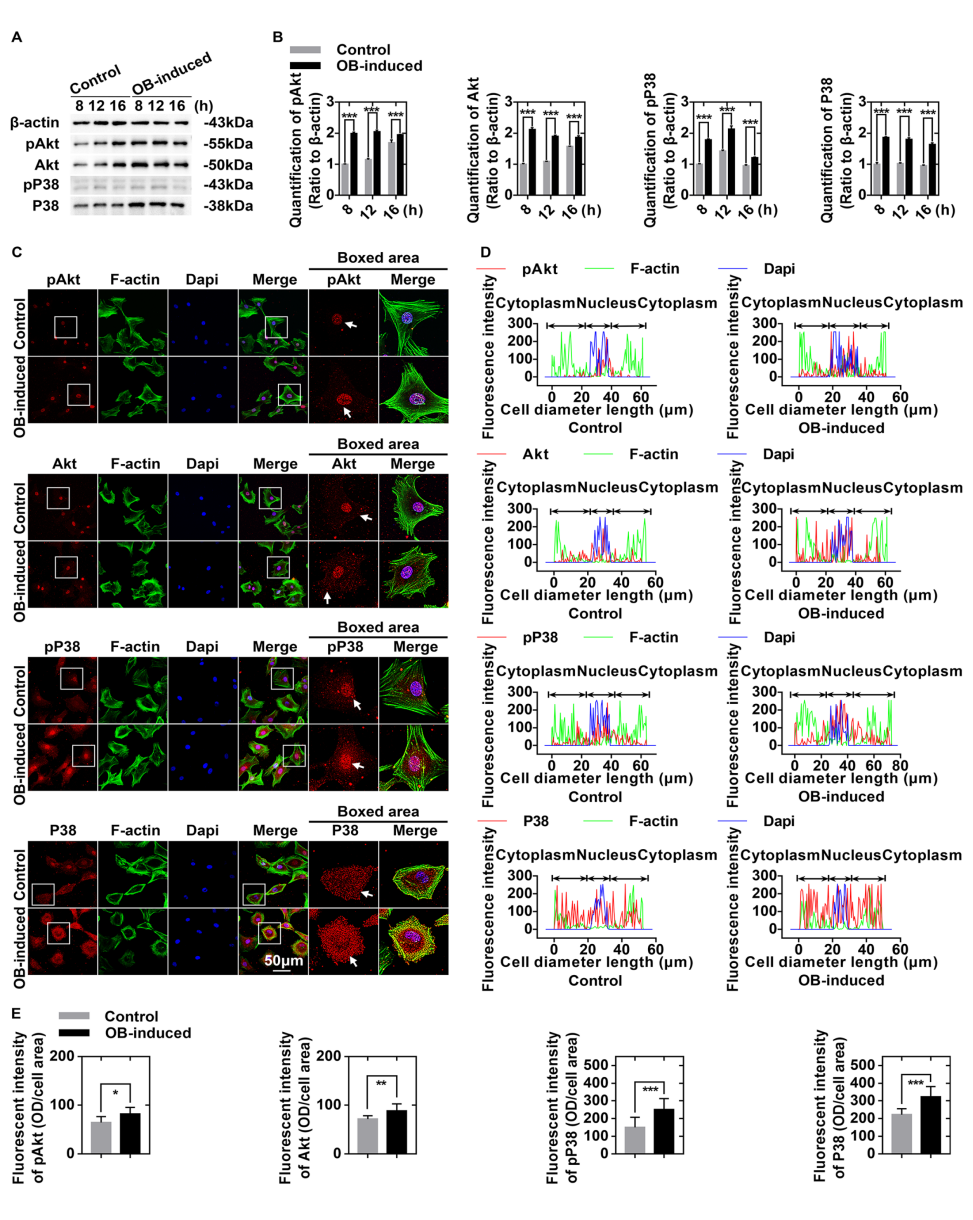
Figure4 Osteoblasts regulate glucose-derived ATP production through the Akt and P38 signaling pathways in chondrocytes (A) Western blots showing the upregulation of pAkt, total Akt, pP38, and P38 in chondrocytes induced by osteoblasts. Cell lysates were collected at 8, 12, and 16 h after co-culture. The gels are representative of three independent experiments (n=3). (B) Quantification was performed to analyze changes in pAkt, total Akt, pP38, and total P38 in (A). (C) Immunofluorescence staining of pAkt, total Akt, pP38, and P38 (red) in chondrocytes after 8 h of co-culture with osteoblasts. The cytoskeletons were stained with FITC-phalloidin (green), and the nuclei were stained with DAPI (blue). The images observed by CLSM were from three independent replicates (n=3). (D) Image-Pro Plus 6.0 was used to determine the linear fluorescence intensity and explore the distributions of pAkt, total Akt, pP38, and P38 in (C). Data analysis was performed on at least 10 cells per group. (E) Fluorescence quantification was performed to show the changes in pAkt, total Akt, pP38, and P38 in (C). The results were from three independent experiments (n=3). The data in B and E are presented as the mean±SD. A significant difference was observed in control chondrocytes. *P<0.05, **P<0.025, and ***P<0.01. OB, osteoblasts.

We performed western blot analysis to show the potential signaling pathways by which osteoblasts regulate glucose-derived ATP production in chondrocytes ( Figure 4A). The protein expression levels of Akt, pAkt, P38, and pP38 were increased (more than 1.750-fold in all members) in chondrocytes, especially at 8 h after co-culture with osteoblasts ( Figure 4B). Immunofluorescence staining revealed that Akt, pAkt, P38, and pP38 were accumulated both in the cytoplasm and in the nuclei, and their expression levels were higher in the osteoblast-induced chondrocytes than in the control chondrocytes ( Figure 4C). Fluorescence quantification confirmed these changes in expressions of Akt, pAkt, P38, and pP38 ( Figure 4D,E).

## Discussion

Homeostasis within the bone-cartilage unit relies on appropriate and dynamic intercellular communication between chondrocytes and osteoblasts [ 4– 6]. Alterations of energy metabolism in chondrocytes are observed in the onset and progression of OA [26]. However, what remains unclear is whether osteoblasts could induce changes of energy metabolism in chondrocytes. Therefore, this study was designed to demonstrate for the first time that osteoblasts induce glucose-derived ATP perturbations in chondrocytes through noncontact communication. Our results showed that in osteoblast-induced chondrocytes there exists an energetic shift characterized by enhanced glycolysis and impaired tricarboxylic acid cycle, which is probably mediated by the activation of Akt and P38 signaling pathways.

The metabolic flux that occurs with TCA cycle is affected in osteoblast-induced chondrocytes. First, a decrease in the number of mitochondria was observed in osteoblast-induced chondrocytes ( Figure 1D,E). Second, the expressions of enzymes (Got1 and Cs) in the TCA cycle were decreased in osteoblast-induced chondrocytes (Figures 2 and 3). Got1 participates in the anaplerotic reaction of the TCA cycle; thus, the decrease in Got1 probably indicated the undersupply of intermediate metabolites for the TCA cycle. Meanwhile, Got1 is the key enzyme in the malate-aspartate shuttle (MAS) that transfers electrons associated with NADH from the cytoplasm to the mitochondria [27]. In this study, the downregulation of Got1 indicated the impairment of MAS, which also appeared to preserve mitochondrial respiratory capacity via downregulation of TCA cycle intermediates [ 28, 29]. In addition, Cs is the entry enzyme into the TCA cycle and the crucial biomarker associated with mitochondrial content [30]. The decreased expression of Cs showed high concordance with the decreased mitochondria and compromised TCA cycle in osteoblast-induced chondrocytes [ 31– 33]. Furthermore, alterations in the TCA cycle probably suggested a deviation from oxidative phosphorylation to anaerobic glycolysis in chondrocytes after 3 days of co-culture with osteoblasts [10]. Indeed, we observed increased expressions of glycolytic enzymes (Gpi, Pfkl, Aldoc, Gapdh, Tpi1, and Pgk1) ( Figures 2 and 3) and increased secretion of the glycolytic end product (lactate) in the osteoblast-induced chondrocytes ( Figure 1C). These results indicate enhanced glycolysis in chondrocytes after 3 days of co-culture with osteoblasts. The increased accumulation of secreted lactate facilitates the regeneration of NADH and reduces oxidative stress, thus contributing to the sustainability and acceleration of glycolysis [23].

There has been a growing concern about the centre-stage role of ATP in metabolism and signaling. ATP provides thermodynamic driving forces for various cellular processes, such as extracellular matrix synthesis and organelle transport [ 34, 35]. In addition, ATP acts as a phosphate-group donor, an adenylyl-group donor, or a coenzyme for substrate activation, post-translational modifications or initiation of enzymatic reactions. ATP is also indirectly involved in signaling via the second messenger, and directly involved in signaling as the ligand of G-protein coupled, ATP-sensitive or purinergic ionotropic receptors [36]. The balance between ATP production and consumption in chondrocytes is critical for extracellular matrix turnover and cartilage homeostasis. Notably, chondrocytes are submitted to complex mechanical stimuli in joints, which induce the export of intracellular ATP into the extracellular environment. For example, various mechanical stimuli trigger ATP release in a way dependent on the Piezo1 ion channel in chondrocytes [37]. Then ATP release mediates the coupling of the Piezo1 channel and purinergic P2 receptors to transduce mechanical cues and facilitate functional regulations [38]. Additionally, chemical signals are also involved in the process of ATP release. For chondrocytes, transforming growth factor-β1 was observed to induce ATP release, while insulin-like growth factor 1 attenuated extracellular ATP accumulation [39]. Emerging evidence suggests that disturbed ATP homeostasis or ATP-based signaling results in the incidence and aggravation of diseases including OA. The reduction of intracellular ATP level in OA chondrocytes is accompanied by the diminishment of ATP release into the extracellular environment [ 26, 40]. This study mainly explored intracellular ATP perturbations in chondrocytes in order to provide potential therapeutic targets for OA. However, it should be admitted that extracellular ATP plays a pivotal role in the regulation of cell functions and intercellular communication via autocrine and paracrine signaling [38]. ATP release in vivo is more complicated, as cells are embedded in 3D peri- and extracellular matrix, affected by neighboring and distant cells, and regulated by various biological, biochemical, and biomechanical signals [ 41, 42]. Therefore, further research is needed to illuminate the potential alterations of ATP release and relevant mechanisms in osteoblast-induced chondrocytes.

The increase in ATP generation is based on the total effect of the compromised TCA cycle and the promoted glycolysis in osteoblast-induced chondrocytes. Since the flux occurs with the TCA cycle and oxidative phosphorylation generates approximately 36 ATP molecules per glucose molecule, glycolysis has been considered to be less efficient, with a net production of 2 ATP molecules per glucose molecule [11]. In osteoblast-induced chondrocytes, the compromised TCA cycle and the promoted glycolysis indicate a switch to a more glycolysis-dependent and energy-inefficient phenotype [43]. However, ATP output is not only insusceptible but also increased in osteoblast-induced chondrocytes ( Figure 1B). Therefore, compared with the inhibition of the TCA cycle, glycolysis was enhanced on a much larger scale in chondrocytes after co-culture with osteoblasts.

Glycolysis is a quick process that accounts for a large percentage of ATP production in chondrocytes under physiological conditions. Although the research results varied, most studies supported that chondrocytes produce 60%–80% of the total ATP through glycolysis [ 44– 46]. Moreover, due to the hypoxic microenvironment, low-content mitochondria, and impaired electron transport chain, the ATP produced in the TCA cycle is only one-fourth of the total ATP in laboratory-cultured chondrocytes. Considering the much lower oxygen tension in articular cartilage than that under laboratory conditions, the ATP generated through the TCA cycle could be negligible *in vivo* [47]. It was revealed that ATP production of mitochondrial respiration accounted for less than 3% of the total in bovine articular chondrocytes [48]. However, as the result of monolayer expansion and mitochondrial biosynthesis, this proportion went up to 36% within one week and continued to increase over time. Meanwhile, approximately a 2-fold increase in lactate production rate and a 30-fold increase in oxygen consumption rate were also observed in this process. These findings indicate a shift from glycolysis to mitochondrial respiration in chondrocytes after monolayer expansion, which is probably related to increased energy-consuming activities in vitro, such as extracellular matrix synthesis and ionic homeostasis maintenance [48]. In rabbit articular chondrocytes, monolayer expansion for six days induced a 13-fold increase of mitochondrial DNA, but the increased mitochondrial DNA was not in coordination with nuclear DNA to code for mitochondrial respiratory chain complexes [49]. The increasing rate of mitochondrial activity was lower than that of mitochondrial DNA content [50]. These studies undisputedly showed that chondrocytes altered their metabolic phenotypes in monolayer cultures. On the contrary, 3D models provide chondrocytes with a better-organized biomimetic microenvironment with integrated biochemical and biomechanical signals, and are privileged to maintain chondrocyte phenotypes and functions [51]. It was reported that 3D models conferred cells with reduced mitochondrial activity and enhanced glycolytic metabolism [52]. However, we still determined to establish a monolayer culture system for its simplicity, applicability, efficiency and inexpensiveness. In monolayer cultures, cells are cultured in the same plane with identical conditions, since soluble factors are distributed homogeneously and exchanged freely in culture medium. Moreover, cells in monolayer cultures can be monitored, screened, and collected in a simple way [53]. In our study, early-passage chondrocytes (at the first two passages) were used to minimize cellular phenotypic alterations during monolayer cultures in the transwell co-culture system [ 54, 55]. Direct intercellular communication is inhibited because the transwell chambers physically separate chondrocytes from osteoblasts. However, chondrocytes and osteoblasts could interact with each other via paracrine ways, since soluble factors they released, such as vascular endothelial growth factor B, platelet-derived growth factor, fibroblast growth factor and bone morphogenetic protein, are allowed to cross the porous membranes of transwell chambers [ 17, 56]. Therefore, we deduce that osteoblasts allow chondrocytes to metabolize glucose by a method more similar to that of the physiological conditions via noncontact communication; thus, the total energy output relies mainly on glycolysis instead of the TCA cycle in chondrocytes. This would provide a theoretical basis for the application of chondrocyte-osteoblast co-culture system in osteochondral tissue engineering [57].

**Figure 5:**
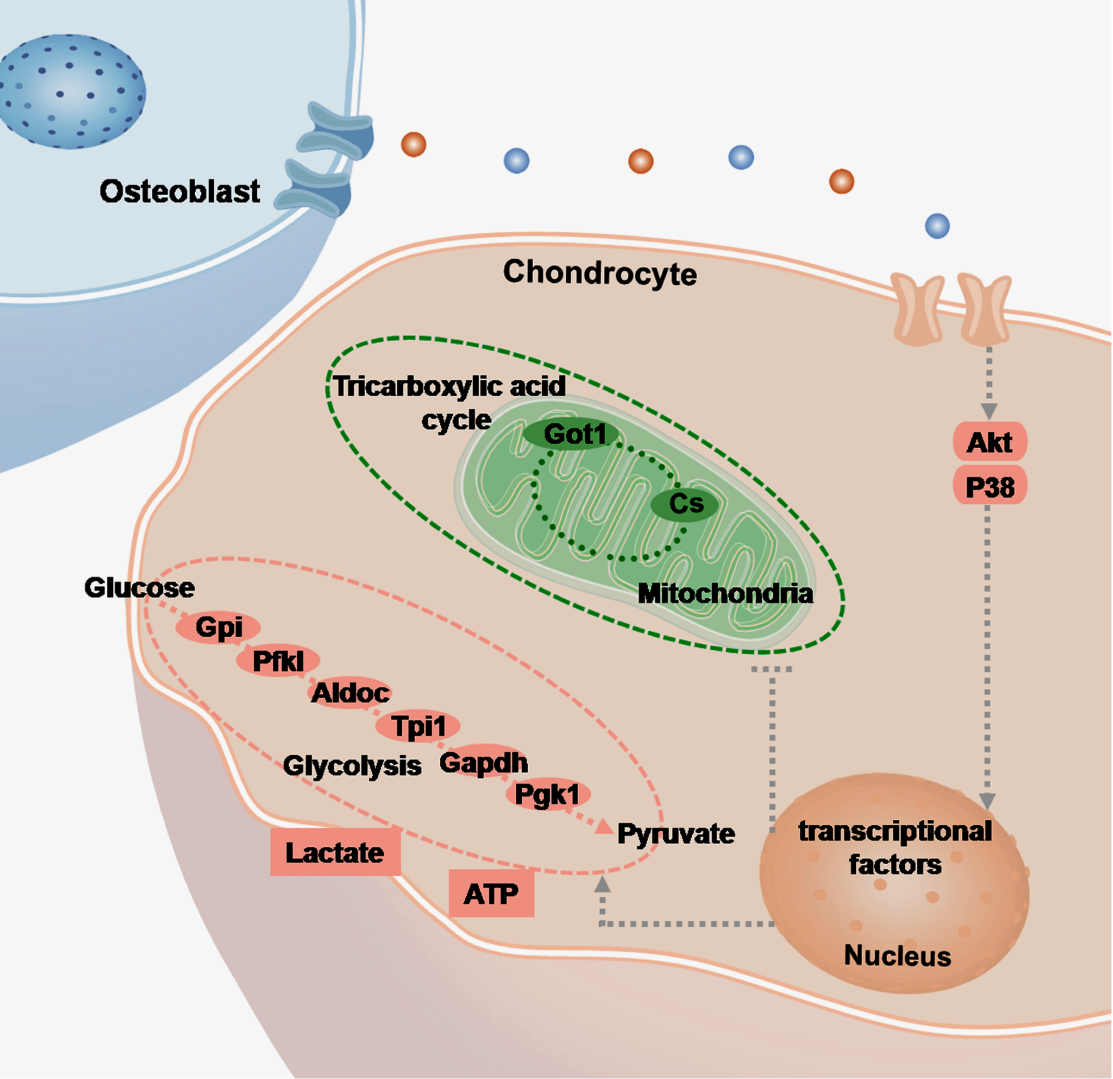
Figure5 Schematic diagram illustrating the changes in the flux of glucose-derived carbons for ATP production in chondrocytes induced by osteoblasts Osteoblasts activate Akt and P38 signaling pathways in chondrocytes via noncontact communication (shown in pink rounded-rectangle boxes). The grey dotted lines point to the possible downstream targets identified by existing studies. Dramatic changes in the flux of glucose-derived carbons for ATP production are induced. The expressions of glycolytic enzymes (Gpi, Pfkl, Aldoc, Tpi1, Gapdh, and Pgk1) are increased, while the expressions of enzymes in the TCA cycle (Got1 and Cs) are decreased (shown in pink and green ellipses, respectively). In osteoblast-induced chondrocytes, although the TCA cycle seems to be suppressed (encircled with green dotted lines), glycolysis is promoted (encircled with pink dotted lines); thus, intracellular ATP level is significantly increased (shown in pink rectangular boxes).

Complicated molecular mechanisms are involved in significant changes in the flux of glucose-derived carbons for ATP production. In our transwell co-culture system, potential soluble paracrine factors activate the Akt and P38 signaling pathways in the early stage of co-culture ( Figures 4 and 5). Activated Akt is associated with increased ATP level [ 58, 59]. The activation of the Akt signaling pathway induces enzymes and transporters in the process of glycolysis, such as 6-phosphofructo-2-kinase/fructose-2, 6-bisphosphatase [60], lactate dehydrogenase A [61], glucose transporter 4 [ 62– 64] and monocarboxylate transporter 4 [61]. These effects are directly involved in the increased glycolytic flux. Akt also induces glycolysis via hexokinase. Hexokinase is the first rate-limiting enzyme of glycolysis, which keeps glucose inside the cells by phosphorylation. Akt regulates the interaction between hexokinase and mitochondria to promote the flux of glucose-derived carbons for ATP production via glycolysis. After the induction of glycolysis, Akt also indirectly regulates the TCA cycle through mitochondrial shuttles [ 65, 66]. Additionally, Akt could rewire cellular metabolism by some transcription factors [67]. For example, hyperactive Akt can phosphorylate and inactivate the transcription factor FoxO to induce the expressions of glycolytic genes [68]. In addition, activation of Akt facilitates the abundance of HIF1α [ 69, 70]; thus, nearly all glycolytic enzymes are then activated, and the TCA cycle is suppressed [71]. Similarly, we found that the downstream targets of the P38 signalling pathway are probably related to the changes in the flux of glucose-derived carbons for ATP production. Previous studies have revealed that P38 may play a positive role in the plasma membrane localization of glucose transporters to increase glucose uptake [72]. Meanwhile, activation of P38 could upregulate glycolytic enzymes, such as hexokinase and 6-phosphofructo-2-kinase/fructose-2, 6-bisphosphatase, in a direct or indirect way [ 73– 75], and the expressions of genes in the TCA cycle were found to be reduced via the P38 signaling pathway [76]. The P38 signaling pathway is also important to preserve the protein stability and transcriptional activity of HIF1α [77], which may lead to the downregulation of the TCA cycle [78].

In summary, this study shows that osteoblasts induce more ATP generation in chondrocytes through an energetic shift characterized by enhanced glycolysis and impaired mitochondrial TCA cycle. Upregulation of the Akt and P38 signaling pathways is probably involved in the coordination of osteoblast-chondrocyte crosstalk to mediate ATP perturbations in chondrocytes. The results of this study may deepen our understanding of the maintenance of metabolic homeostasis in the bone-cartilage unit. However, further research is required to provide more insight into the potential mechanisms of osteoblast-chondrocyte metabolic interactions.
